# Daytime sleepiness in Chinese professional, semi professional and students soccer players in the Shanghai lockdown

**DOI:** 10.1186/s13102-023-00730-3

**Published:** 2023-09-22

**Authors:** Songhui You, Antonio Cicchella

**Affiliations:** 1 Tongji University, International College of Football, 1239 Siping Road, Shanghai, P.R. China; 2 Department for Quality-of-Life Studies, University of Bologna, Corso d’ Augusto 237, Bologna, Rimini, 47921 Italy

**Keywords:** Daytime sleepiness, Fatigue, Stress, Female soccer players, Epworth Scale

## Abstract

**Purpose:**

April-May 2021 Shanghai city was under strict lockdown. Soccer players suffered from the restrictions, being unable to train and have a social life. The aim of this study was to compare differences in daytime sleepiness between genders and qualification levels in a cohort university Chinese soccer player under 0 Covid policy restrictions in the urban area of Shanghai.

**Methods:**

491 questionnaires of Epworth Sleepiness Scales (ESS) were compiled online by male and female Soccer Students (SS), Semi-Professional players (SP) and Professional Players (PP) during the ongoing restriction measures post Shanghai lockdown. ANOVA was performed for players levels and gender.

**Results:**

Significant differences were found between the 3 levels and between males and females. PP showed a very low score in the ESS (5,97) well below the threshold of 8 for daytime sleepiness, while SS and SP showed an ESS score above the threshold. Female showed higher scores in comparison to males. Differences between males and females reflect the confinement conditions in the ESS items, showing more difference in the item of ESS which are related with indoor situations.

**Conclusions:**

This study shows the first data on sleepiness in Chinese soccer players of different level of qualification immediately post lockdown condition. Professional male’s players sleepiness was lower, than females, SS and SP after the exceptional lockdown measures. The reasons can reside in the more ordered lifestyle of PP in comparison to SS and SP, which mitigated the effects of the lockdown. Our results suggest that measures to improve sleep in females’ soccer player should be adopted if these exceptional conditions will happen again.

## Introduction

After decades of focusing merely on the performance, there is today more public awareness about the wellbeing and quality of life of the athletes and consequently research in the field has increased [1]. This is a shift of paradigm in sport science, that put the athlete’s health at the center. Studies on athletes’ well-being in sport performance has been thus increased, to ameliorate the health of the athlete/worker. Among the parameters who affect the quality of life, sleep has a significant role. Sleep is a determinant of several illnesses and relates to several psychological and physical diseases [2], not to mention the effect of sleep on performance [3, 4]. It has been shown that poor sleep in soccer players negatively affected athletic and match performance while also increasing the number and severity of musculoskeletal injuries [3], while good sleep (> 8 h) is an effective way to recover and improve the performance [4].In athletes, poor sleep quality has been reported more by female athletes than by male athletes: 31.4% of men and 48.8% of women had poor subjective sleep quality [5] and females experience diminished total sleep times [6]. Elite athletes, seems to have shorter sleep than lower-level athletes [7, 8] because of higher hormonal activation (adrenal axis arousal), and higher social pressure [9]. At least in western players, sleep disruption has been associated with social pressure, caffeine usage [10], and high intensity training [9] has been related to poor sleep. Forced exercise has been shown to cause circadian phase shifts in the kidney, liver, and submandibular glands by activating the sympathetic nervous system and the hypothalamus-pituitary-adrenal gland axis [11]. These circadian shifts can have consequences on the individual health causing diseases on liver (glucose and insulin imbalances) and kidney (overload with subsequent glomerular failure) [11]. These damages happen by means of the impairment caused by excessive exercise on the central nervous system, which is ubiquitous in the human body, thus targeting many organs. Considering sleep, has been observed that, after prolonged exercise, the circadian (temporal rhythm in functioning) phases in the kidney and liver appeared accelerated in mice [11] dealing to an overload on these organs. A recent study, showed a role for lactate in possible damages to the kidney in humans, possibly associated with other stressors, such as heat or poor recovery (included sleep) [12]. Beside these factors, it has been hypothesized that Elite athletes can possess personality characteristics which predispose to success in elite sport (e.g. perfectionism and anxious concern) and predispose individuals to sleep disturbances [13].

The Covid-19 Lockdown also impacted athletes training [14, 15] and powerful impacted lifestyles. In Europe, it seems there weren’t worsening of the performance or an increased rate of injury [15]. In fact, it was observed that the development of physical match performance from pre to post COVID-19 break in Italy, Spain and Germany [15], differed between professional European soccer league. In the German Bundesliga, parameters of physical match performance remained unaffected while psychological and mood states were altered in male and female professional and non-professional soccer players, by the restriction imposed by Covid 19 epidemic, increasing depression and anxiety [15]. Italian Serie A showed a decrease in most parameters of physical match performance after the COVID-19 break [15]. Sleep quality and length has also been affected significantly by the lockdown. In a study performed on 175 Spanish football players of different level, comparisons between gender showed men slept more hours before the isolation period than women and had better sleep quality than women during the isolation period [14]. Thus, more than physical conditions that can be in a certain extent maintained with a proper training, the effect of lockdown was mostly psychological.

Shanghai complete lockdown was a unique setting for studying sleep related behaviors. It lasted more than 2 months and continued for several months with intermittent opening-closing of public places and sport facilities, and suspension of sport events. Universities and other large communities were locked when some positive cases were found. As normally the universities in China are organized as closed compounds, confinements in dormitories and limited mobility, were applied, and food delivery was organized. University students has been especially affected by lockdowns because of the tightest restrictions in the campus, in comparison with the outside environment. Shanghai is a megapolis with an estimated population of 27 millions of habitants. Tongji University, in Siping Campus of Shanghai Yangpu district, has a large College of Football, which enrolls both professional (dual career), semi-professional and student players, which were double affected by the restrictions: as students and as players. This fact can have altered players mood and thus influenced sleep habits. A study performed during lockdown in England on high level athletes, showed that during lockdown 20% of the dual-career athletes adopted a negative coping strategy with the lockdown measures [16] but the length of the lockdown was shorter than in Shanghai and the restrictive measures looser. In fact, Shanghai lockdown has unique features, due to the implementation of restriction never applied worldwide, which deeply impacted the society at all levels, and athlete’s life too.

There is some evidence among professional soccer players that males had a better sleep quality than women during the isolation period [14]. However, also in normal life conditions, sleep disturbances in females’ soccer players has been only recently studied [9], showing a high incidence of sleep disturbance associated with acute factors such as the pressure of the night pre-game [17], post-game or post training [18] with a decrease in sleep time in those conditions. Female sleep has been shown also to be sensitive to menstrual cycle and hormonal imbalances [19] which in turn affect arousal’s control, stress response, and memory consolidation [19]. However, the details of female physiology during sleep are not yet clear, and further studies are necessary to better understand the increased propensity for mental health disorders linked to sleep disturbances in females [19]. From the response of a survey in 231 woman (non-athletes, 18–28 years old), it was revealed that during the 2021 lockdown, the habit of going to bed at night after 12 p.m. was significantly increased [20] and was associated with disturbances in menstrual cycle. Another study performed in lockdown condition in China on 2084 university students, reported 10% higher rates of sleep disorders in females than male students. Female also experienced a higher incidence of mental disorders having as a mediating factor sleep [21]. However, findings about differences between male/female in sleep are controversial in different sports. A study in 146 top Brazilian Olympic athletes of various sport showed worse sleep (measured with polysomnography) in man than in woman [22], however this study didn’t investigate soccer players, which normally are subjected to higher pressure at high level. The same study showed that 36% of all athletes had a sleep disorder with a greater reduction in sleep quality in men than in women. Female athletes can also show a higher increase in inflammatory biomarkers and a higher fasting insulin level subsequently to sleep disturbances in comparison to males [23]. A higher circulating cortisol, which is known to increase as a response to sleep loss, has been observed after sleep deprivation in sport in comparison to non-athlete females [24]. In another study comparing professional and Olympic athletes, was found that Olympic athletes has less disturbances than recreational athletes, but in the sample weren’t professional soccer players [25]. Daytime sleepiness was found in 22.5% of a sample of 111 Qatar male professional players [26]. Data on sleep after a prolonged lockdown in professional players are becoming now available [14, 15] but at our knowledge any data exist on daytime sleepiness in “normal” life conditions and during and after a lockdown in Asian male and female soccer athletes of different level of qualification. Albeit a few studies exist on sleep during lockdown in professional players [14, 15], there are any data on daytime sleepiness in females’ players and in players of lower level of qualification and no data exist in collegiate players. Shanghai lockdown underwent unparalleled restrictions and was an exceptional condition to study daytime sleepiness in soccer players. Aim of this study was to compare differences in daytime sleepiness between genders and between qualification levels in Chinese soccer players of different level of qualification after the 0 Covid policy restrictions.

## Methods

Epworth sleepiness scale (ESS) is an 8 items clinical scale commonly used for the measure of sleepiness [27, 28] and has been extensively used in different cultural contexts [29, 30], and in clinical and sport populations [31]. It has the advantage of being easily administrable, to have extensive normative references [32], and to be available in several languages [33–36]. The ESS was validated also for Chinese language [37, 38]. It comprises 8 items asking how the subject feel sleepy in different daily living activities concerning life situations while a person can feel sleepiness during the day, can be answered in a 0–3 scale [37]. The ESS shown to be stable in time across a period of one year [39] and consistent with other questionnaires for sleepiness [40]. A score of > 8 in the ESS is the threshold for the diagnosis of daytime sleepiness [5].

1200 ESS questionnaire were submitted online. A list of subjects was prepared, and the targeted subjects were recruited by mean of university offices, by direct contact, trough the local football federation, and mainly with the social media Wechat. Subjects were invited to fill the Chinese version of the questionnaire built in the Chinese social media Wechat (Tencent Technologies, Shenzen, China) [41]. The student population was almost the totality of Tongji University International College of Football while the semiprofessional and professional football players were enrolled in the Chinese League at various level from different cities in China and were personally invited to fill the questionnaire. The questionnaire was send to the total Tongji University College of Football females’ students consist of 60 subjects and the response rate was 83% for this group. All the subjects lived in the campus in the same life conditions and undergone the same restrictions. The subjects originated from different parts of China, but lived in Shanghai. The subjects were identified with the help of campus services and represented the totality of the soccer players population. In the lockdown, training was restricted to individual training and no group training was possible. Players lived in the dormitory, with possibility to exit only in the dormitory garden, and went out once a day for the mandatory Covid-PCR test. The questionnaire was submitted immediately after the thigh lockdown measures, with some restriction still going on. Wechat has proven to be a valuable tool to submit online questionnaires and in China is used by everyone [42]. The questionnaires were sent to all Wechat address of the students and was completed by 3 different groups: soccer students of Tongji University International College of Football (SS), semi-professional (SP) and professional players (PP) in the city of Shanghai in the weeks 16 to 31 October 2022, with still ongoing limitation to mobility. During the filling period, a 10-day complete closure of the campus was imposed. Shanghai experienced a complete lockdown from 28 of March till 30 of May 2022, thus the questionnaire was filled 4 months after the complete lockdown with some restriction still going on. All the subjects, including the professional players, were also university students. Players’ levels were defined according to the rules of CFA (Chinese Football Association), which are complex, but guarantee a tight classification because are grounded in several performance parameters (e.g., number of games in one year in the division) and on the career of the player. SS were students in the International College of Football of Tongji University and participate in soccer training at least four times per week plus an inter-university weekend match, while SP and PP trained once per day, SP play a regional match every weekend, and PP play a match every 4 days, and sometime twice per week in the major Chinese League, traveling all along China. The measures were performed during the championships season, but of course no games were possible, due to the restrictions. The study was conducted according to the guidelines of the Declaration of Helsinki and approved by the Ethics Committee of Tongji University (approval code: tjdxsr029). Informed consent was obtained by the participants, after the explanation of the aim of the study.

Gender and level comparisons was performed using One way ANOVA (Scheffe’ post-hoc) with SPSS v.20.0 and significance level was set a 0.5. Post-hoc statistical power with an MDE (minimum detectable effect) of 0.5, was calculated at 0.9732. Effect size was 1.31–8.58 (p = 0.000).

## Results

A total of 491 soccer players (response 40.91%), 441 male and 50 females filled the questionnaire. Mean age for the total group was 19,16 ± 2,68 years (19,17 ± 3,41 for males and 19.10 ± 1.68 for females. 407 were soccer collegiate students (110 from Tongji University, 60 males and 50 females, 83.3% of all female soccer players in Tongji University) (SS), 55 were semi-professional (SP, Level 1 and 2 CFA) and 29 were professional players, playing in the Chinese major league (PP). Among females, 21 were SS and 29 SP players, among males, 386 were SS and 56 SP players. Data were all normal distributed at Kolmogorov-Smirnoff test. Descriptive statistics are summarized in Table 1.

**Table 1:** Epworth scale for the different groups (SS = soccer students, SP = semiprofessional, PP = professional).

Level	Gender		Age (years)		Epworth Score	
SS	Males	Mean	18.49		7.89	
		N	386		386	
		Std. Deviation	5.14		3.45	
	Females	Mean	18.24		9.05	
		N	21		21	
		Std. Deviation	1.37		3.35	
	Total	Mean	18.48		7.95	
		N	407		407	
		Std. Deviation	5.07		3.45	
SP	Males	Mean	20.31		9.85	
		N	26		26	
		Std. Deviation	1.89		4.81	
	Females	Mean	19.72		10.14	
		N	29		29	
		Std. Deviation	1.2		4.28	
	Total	Mean	20		10	
		N	55		55	
		Std. Deviation	1.76		4.50	
PP	Males	Mean	27.21		5.97	
		N	29		29	
		Std. Deviation	3.44		3.05	
Total	Males	Mean	19.17		7.88	
		N	441		441	
		Std. Deviation	2.77		3.58	
	Females	Mean	19.10		9.68	
		N	50		50	
		Std. Deviation	6.81		39.20	
Total		Mean	19.17		8.07	
		N	491		491	
		Std. Deviation	2.68		3.65	

Age frequency is reported in Table 2.

**Table 2:** Age frequency in males and females

Males			Females	
Age (years)	Frequency		Age (years)	Frequency
16	4		16	1
17	24		17	6
18	225		18	18
19	86		19	5
20	43		20	8
21	18		21	7
22	7		22	4
23	4		23	1
24	3			
25	5			
26	2			
27	2			
28	2			
29	12			
30	2			
31	2			

Anova (Scheffe post-hoc) results are reported in Table 3.

**Table 3:** Differences between genders and qualification levels

Variable	df	Nr.	F	p
Age/Levels	2	490	361.42	0.000
Total ESS score/Levels	2	490	13.36	0.000
Total ESS score/Gender	1	490	13.02	0.000
Item 1/Gender	1	490	9.76	0.002
Item 2/Gender	1	490	11.88	0.001
Item 3/Gender	1	490	11.22	0.001
Item 4/Gender	1	490	13.02	0.000

Item 1: “sleeping while sitting and reading”; Item 2: “while watching television/mobile”; Item 3: “sitting and talking with someone”; Item 4: “sitting quietly after a lunch without alcohol.”

Total ESS score was significantly different between the 3 levels of qualification. Also, age was significantly different between the 3 levels, with PP being the elder.

Four items of the Epworth scale were found different between males and females. Females declared to feel sleepier in the following items: “sleeping while sitting and reading” and “while watching television/mobile” “sitting and talking with someone”; “sitting quietly after a lunch without alcohol”. Total score was also significantly different between the genders .

Interestingly, the items which resulted different between genders, concern indoor situations, albeit ESS has also item referring to actions happening in an outdoor setting.

The percentages of subjects over the threshold of daytime sleepiness (ESS > 8) were : SP females (72%) ; SP males (65.8%) ; SS females (52%) ; SS males (41.6%). The overall SP group (male and females) showed a 69.09% of subject over threshold and the overall SS group 42%. In the overall group females showed the higher % of subject over threshold (64%) while males had 43.20% above the threshold for sleepiness. We found a percent of above threshold sleepiness subjects in SP dramatically higher than those found in a sample of professional players in Qatar 22.5% (ESS > 8) [26].

## Discussion

Novelty of this study is in evidencing how females and lower-level players suffered more of the restrictions in comparison to professional players. The study is original because this is the first study in Chinese professional players measruing daytime sleepiness after the exceptional condition of the Shanghai lockdown. Surprisingly, in the lockdown condition our sample of male’s professional players show a very low score of daytime sleepiness contrary to what reported in most of the we reviewed for western soccer players in normal conditions [5, 7, 8, 22]. However, no data exist about sleepiness in Chinese soccer players in “normal” life conditions to compare. A cause can be the higher arousal in these athletes caused by intensive training and playing, or to genetic factors, e.g., subjects with less sleepiness has more chance to reach the top level. Cultural factors can also play a role in explaining the sleep behaviors. In respect to lockdown conditions, it can be hypothesized professional athletes had a more disciplined sleep, in comparison to students. In fact, there is some evidence in the literature about the capacity of professional players to better manage their sleep patterns [43]. Psychoactive nervine substances, such caffeine, can also play a role in diminished sleepiness in western players. It is known that caffeine in China is not diffused like in western world. The present study was performed during the Shanghai lockdown, with restrictive measures going on. At time of filling the questionnaire, the university students and players were completely locked in their dormitories for 10 days while the outside world benefitted of more loose measures. This factor can have significantly influenced the results, increasing the stress levels and thus the sleepiness of the students. Daytime sleepiness has been also related to depression [44], and this is the case of lockdown. In a large sample of children and adolescent the impact of lockdown on mental health has been shown to be dramatic [45]. However, social support was shown to play an important role in reducing the negative effect of lockdown on mental health in 675 non-professional soccer players in China [45], albeit this sample was not investigated for sleep. Cultural differences can also play a role. In Chinese culture, students’ study is highly valued and the competition high, so students may show hopelessness for poor academic performance caused by the lockdown [46].

Despite the controversial literature about sleep disturbances in females’ athletes [16–18, 22] the results of the present study support the presence of a higher percent of sleepiness in female soccer players compared to males. This result is consistent with another study in Spanish professional football players performed during a lockdown, who showed men having better sleep quality than women during the isolation period [14]. However, this latter study didn’t consider daytime sleepiness. The questionnaire captured 83.3% of all Tongji University female soccer players population (60 females’ players in total), however, the number of females is low in comparison to males. The reason for this underrepresentation of females is attributable to cultural factors. Female soccer is just at is beginning in China, and soccer is still perceived as an only male’s sport, thus the results of the present study added a significant knowledge to Chinese female soccer.

Sleep is an emerging as a key factor for health of female players, and it was indicated among the factors to be improved to increase female soccer players health [47]. It remains a question why females are so dramatically affected by sleepiness in comparison to males, due that training status should different and adapted. One hypothesis which is consistent with the available literature is dual career female athletes faces more challenges than males [48] both socially than physically, and the demands faced by players increases as their level of education/vocation becomes higher. This factor can explain the differences found in sleepiness between males and females SP (dual career) players.

One reason can be the training methods for soccer are traditionally developed for men and are less suitable for women, especially in respect to injuries [49] and this can cause a training overload in females, which deals to poor sleep [47, 48]. In fact, as mismatch between the request of the coaches and the capacity of players for training loads has been showed in professional male’s soccer players [5, 50] and can be a causal factor of poor sleep, at our knowledge, no studies exist in females on this topic. Also, psychological-affective reasons (more involvement in their role by females) can influence this outcome.

Professional female soccer players, show higher values for stress control, motivation, mental ability, and team cohesion in comparison to female amateur [51] which, albeit positive traits, can make them more susceptible to emotional distress and exhaustion. Females’ soccer players were found more sensitive to performance evaluation by coaches and peers [35] and more at risk for anxiety and depression than males [52] and to have shorter sleep-in comparison to other professional’s sport female athletes [53]. In normal conditions, the sleep disruption is likely to be caused by the high physical and mental loads experienced during soccer games [54]. Sleep phases changes during night in an alternate way from light to deep and rem sleep [55] Sleep modification in normal conditions (not in exceptional circumstances such as lockdowns) shows an association of deep and REM sleep with fatigue with increased restoring sleep two to three days after match, a pre-match reduction in sleep length [54]. The results of the present study are in agreement with another study, who showed in Italian female professional soccer players an increase in depressive symptoms and a sleep worsening during the lockdown [55]. Interactions between coach/trainer and player were more frequent (i.e., daily) among professional (27%) than amateur (11%) and semipro (17%) players during lockdown [56]. This factor can also have influenced mood and thus less sleepiness in PP. Another factor not investigated in the present study, is the chronotype of the athletes, or the preference for morning or evening activities. In fact, it has been shown that athletes tended to select and pursue sports that suited their chronotype [57].

## Conclusions

The originality of this study resides in the exceptional conditions in which it was performed. Albeit four months after the end of the complete lockdown of the city of Shanghai, this study was performed in very exceptional conditions, never happened before, thus comparisons with other studies are difficult. Further, sleepiness was never assessed in Chinese soccer players, and, at our knowledge, any previous data exist in literature. This study found different behaviors in Chinese in daytime sleepiness in comparison to data in western populations of soccer players presented in literature. Chinese professional soccer players, albeit undergone the same restrictions of the other players, showed a very low score in daytime sleepiness, and this could be explained with the more organized lifestyles; the commitment to result can have reduced the impact of emotional stressor in professional soccer players. It can be also hypothesized the subjects with a higher arousal reach the top level, or the intensive training and playing in professional make them less sleepy through the activation of hypothalamic-surrenal axis. The results of the present study confirm females more susceptible to sleep disturbances than males even under condition of limited mobility. Due to the exceptional experimental conditions of the present study (post Shanghai lockdown with still ongoing restriction measures), the excessive sleepiness in recreational and semiprofessional soccer players could be linked to the daily life restrictions and consequent mood disturbance, while these conditions seemed to affect less the professional players who benefit of better life arrangements. Cultural differences could also explain the observed reduced sleepiness in professional Chinese soccer players in comparison to Western players. The role of sleep in recovery is also a complex issue, few investigated, and there is a need for future research to estimate the importance of sleep and to identify influencing factors. This study shows that recovery process was not complete in soccer players.

Beside these findings, the results suggest than interventions are necessary to improve the sleep hygiene in SP soccer players, especially in females because females’ soccer players are targeted by more stressor than males. Further investigations are necessary on the low sleepiness of professional soccer players, e.g. a study performed in non-emergency conditions and especially cross-ethnicity and cross-cultural studies are needed. Chinese high level soccer players can copy better than western players to stressors. A limit of our study is any data exist about sleepiness before lockdown in the same subjects. Also, the present study didn’t measure any physiological data (e.g., circulating cortisol, inflammatory biomarkers and menstrual cycle) to correlate with the questionnaire. Another limitation is the imbalance between the number males and females’ subjects. Causes of daytime sleepiness can be investigated studying sleep quality with polysomnography, but a polysomnographic study in 491 subjects, albeit feasible, is high challenging and beyond our present possibilities. The present study didn’t assess the use of nervine substances by athletes, such as black tea, and the diet. The measurement of depression could have been helpful in better understanding the results. However, due to the special circumstances in order to not further emotionally challenge the subjects, we choose not to administer a depression scale. A follow-up measurement after the recovering of normal life, will be also necessary. Also, we missed a group of professional females’ athletes to compare with males’ professionals. Shanghai lockdown was a unique setting to study the sleepiness in soccer players. The observed modification in sleepiness behaviors can be useful for the adoption of measure to increase soccer players health and well-being, especially in female soccer players. A lockdown is essentially a social phenomenon, and the implications on health are not yet completely understood. The same statement can be done for sleep. Also, a lockdown could happen again in future, and research can help to adopt proper public health measure.

## Data Availability

All data and materials are available on request to the corresponding author.
